# 

**Published:** 1904-10-15

**Authors:** 


					﻿THE
DENTAL REGISTER
EDITED BY
NELVILLE S. HOFF, D. D.S.
Volt WiUt'S P^TPpER 15, 1904.	No. ,o.
PRICE, ONE DOLLAR A YEAR IN ADVANCE.
PUBLISHED MONTHLY BY
SAMUEL, A. CROCKER & CO.,
THE OHIO DENTAL AND SURGICAL DEPOT.
33 to 31) 'Wr. ”tli St., Ciiiciiiriati, O.
Entered at the Postoffice at Cincinnati, 0., as second-class mail matter.
I
				

